# Dietary *Weizmannia coagulans* Strain SANK70258 Ameliorates Coccidial Symptoms and Improves Intestinal Barrier Functions of Broilers by Modulating the Intestinal Immunity and the Gut Microbiota

**DOI:** 10.3390/pathogens12010096

**Published:** 2023-01-06

**Authors:** Masanori Aida, Ryouichi Yamada, Toshiki Matsuo, Itaru Taniguchi, Shin-ichi Nakamura, Takamitsu Tsukahara

**Affiliations:** 1 Science & Innovation Center, Mitsubishi Chemical Corporation, Yokohama 227-8502, Kanagawa, Japan; 2 Mitsubishi Chemical Corporation, Tokyo 100-8251, Japan; 3 Kyoto Institute of Nutrition & Pathology, Ujitawara 610-0231, Kyoto, Japan

**Keywords:** *Weizmannia coagulans*, *Eimeria*, broilers, anti-inflammatory cytokines, intestinal barrier function

## Abstract

To determine the mechanisms by which *Weizmannia coagulans* SANK70258 (WC) supplementation improved growth performance and coccidial symptoms, we assessed the gene expressions and the microbiota compositions in the small intestinal tissues and digestas of coccidium-infected broilers previously given WC or lasalocid-A sodium (AM). WC supplementation significantly upregulated the gene expressions related to intestinal immunity and barrier functions, such as *IL17A*, *IL17F*, *IL10*, *cathelicidin-2* and *pIgR*. Body weights, and *Claudin-1* and *IL10* expressions were positively correlated (r = 0.41, *p* < 0.05 and r = 0.37, *p* = 0.06, respectively), whereas lesion scores of the small intestine and *IL17A* expression were negatively correlated (r = −0.33, *p* = 0.09). The microbiota analysis detected that genus *Alistipes* was more abundant in WC-supplemented broilers than in control, and positively correlated with body weights and *Claudin-1* expression (r = 0.61, *p* < 0.05 and r = 0.51, *p* < 0.05, respectively). Intriguingly, genus *Enterococcus* was most abundant in WC-supplemented broilers and positively correlated with *IL17A* expression (r = 0.49, *p* < 0.05). Interestingly, *Escherichia-Shigella* was significantly more abundant in the small intestinal digestas of AM-administered broilers than in those of control. To summarize, WC supplementation modulated and immunostimulated the microbiotas of broilers, specifically genera *Alistipes* and *Enterococcus*, which led to the improvement of weight gain and coccidial symptoms, without disrupting the intestinal microbiota compositions, as AM did.

## 1. Introduction

Coccidiosis, a disease caused by parasites of the apicomplexan genus *Eimeria* [1], brings about a litany of symptoms in infected animals, from low appetite, to diarrhea and even death in serious cases [2,3]. Although in recent years there has been a steady increase in the number of cases in poultry, where antimicrobial-resistant *Eimeria* spp. have been detected [4], the gold standard for the treatment of coccidiosis in broilers are still coccidiostats [5]. Therefore, the advent of alternative compounds to antimicrobials has been a long-awaited event for the poultry industry.

Probiotics are microorganisms capable of conferring health properties to the host upon ingestion. In the case of *Eimeria* infection, supplementation with *Lactobacillus plantarum* P8 helped to relieve the adverse effects of coccidiosis [6]. *Weizmannia coagulans* (WC), also known as *Lactobacillus sporogenes* [7,8] or *Bacillus coagulans* [7], is a lactic acid bacterium that has been previously used to induce greater body weight and weight gain in broilers [9]. Moreover, Zhang et al. [9] also observed that WC-supplemented broilers experienced a reduction in the levels of pro-inflammatory factors and accretion of short-chain fatty acids and beneficial bacteria *Alistipes* and *Odoribacter*. At these premises, we previously showed that WC supplementation helped broilers to increase their body weights, to lower intestinal lesion scores and to decrease oocyst counts, in the same manner the administration of coccidiostat lasalocid-A sodium (AM) did [10]. We also showed that WC supplementation, unlike AM, induced neither higher *Escherichia coli* counts nor increased *E. coli* ratios. An abundance of *E. coli* would likely reduce weight gain in the broilers, due to its production of lipopolysaccharide, an inflammation-inducing factor [11]. Nonetheless, the mechanisms by which WC supplementation induced weight gain and lowered the numbers of coccidia remained elusive.

Previous work has shed some light on the obnoxious effects of coccidiosis on the immune response of infected birds. For example, Sharma et al. [12] reported increased production of pro-inflammatory cytokines and permeability of gastrointestinal tracts in 16-day old pullets inoculated with *Eimeria* spp. Separately, Pham et al. [13] also reported that White Leghorn chickens, challenged with *E. tenella*, experienced diarrheal episodes and high intestinal lesion scores (e.g., loss of cecal villi, necrosis and hemorrhage), most likely due to the disruption of intestinal barrier functions in the animals, including downregulation of claudin (CLDN)-1, CLDN-3, E-cadherin, occludin (*OCLN*) and Zonula occludens-1 (*ZO-1*), as well as increased levels of fluorescein isothiocyanate (FITC)-dextran, which indicated high intestinal permeability.

The present study aimed to determine the mechanisms by which WC supplementation improved growth performance and reduced the number of coccidial oocysts. Using small intestinal and cecal digesta samples of coccidium-infected broilers from a previous investigation [10], in the present work, target markers, changed by WC supplementation, were narrowed down by an RNA sequencing analysis and evaluated in detail by gene expression analysis. Afterwards, the results of the gene expression analysis were ascertained in relation to the intestinal microbiota.

## 2. Materials and Methods

### 2.1. Sample Collection

The present study was a follow-up work of an investigation using SANK70258 as the experimental WC strain, which has been already reported [10]. Therefore, the small intestinal and cecal digesta samples, as well as the duodenal tissue samples used in the present work, were the same as those from broilers dissected in the previous study [10].

### 2.2. Sample Preparation and RNA Sequencing Analysis

Duodenal tissue samples of 29-day-old broilers were used for the RNA sequencing analysis of the transcriptome profiles because the duodenum is believed to be the main colonization site of *E. acervulina*, and in animals of this age, the numbers of this coccidium were significantly reduced by WC supplementation [10].

Total RNA was extracted from the tissues as follows. The frozen tissue samples, previously soaked in RNA-later solution (Sigma-Aldrich, Tokyo, Japan), were thawed and washed with sterile PBS three times. Afterwards, 50 mg was sub-sampled from each sample. The sub-samples were then finely broken down with a TissueRuptor apparatus (Qiagen, Tokyo, Japan) in 1 mL of TRIzol reagent (Thermo Fisher Scientific, Tokyo, Japan). After the tissues were broken down, solutions were prepared by adding 0.2 mL of chloroform (Fujifilm Wako, Osaka, Japan). The solutions were then centrifuged at 12,000× *g* for 15 min at room temperature. After centrifugation, the supernatants were collected into new sterile microtubes, and equal volumes of 70% ethanol were then added. Next, the supernatants were loaded into columns of a commercial kit (RNAeasy mini kit; Qiagen, Tokyo, Japan). Purification and extraction of the samples were conducted as per the manufacturer’s instruction. During the column purification, DNA was digested with DNAse I (Thermo Fisher Scientific, Tokyo, Japan). Portions of the total RNA solutions were sent in dry-ice to Macrogen Japan (Tokyo, Japan) for further analysis.

Using an Agilent 2100 Bioanalyzer (Agilent Technologies, Santa Clara, CA, USA), it was confirmed that all samples had RNA integrity number values ranging from 6.4 to 8.9. A 100-bp paired-end sequencing was carried out using a Novaseq 6000 System (Illumina, SanDiego, CA, USA). Dry analyses were also conducted at Macrogen Japan, from which fastq files were obtained. The raw sequencing data were evaluated by Fast QC ver. 0.11.7 (available at https://www.bioinformatics.babraham.ac.uk/projects/fastqc/; accessed on 12 July 2022), which confirmed that their quality was good enough to be used for further analysis. Sequences were trimmed with Trimmomatic 0.38 (available at http://www.usadellab.org/cms/?page=trimmomatic; accessed on 12 July 2022), and trimmed reads were mapped according to the reference genome with HISAT2 version 2.1.0 (available at https://ccb.jhu.edu/software/hisat2/index.shtml; accessed on 12 July 2022) and spliced-read mapped through Bowtie2 aligner. The transcripts were then assembled with the aligned reads using StringTie version 2.1.3b (available at https://ccb.jhu.edu/software/stringtie/; accessed on 12 July 2022). The read count values of the known genes, obtained via the StringTie, were used as the original raw data. During the data pre-processing, low quality transcripts were filtered out. Afterwards, the trimmed means of the M-value were normalized. Next, a Differentially Expressed Genes analysis was carried out, and a follow-up statistical analysis of these data was conducted using Fold Change and an exactTest using edgeR. Significant results were selected based on the following conditions: |fc| ≥ 2 and exactTest raw; *p* < 0.05. The resulting data showed that 66 genes met these conditions. For the significant list, a hierarchical clustering analysis was carried out on groups with similar samples and genes. In the present work, the results are graphically shown using a volcano plot. The enrichment test was carried out using a Gene Ontology (GO) term enrichment analysis (http://geneontology.org/; accessed on 12 July 2022), and the significant gene list was compared against the GO database using the g:Profiler tool (https://biit.cs.ut.ee/gprofiler/; accessed on 12 July 2022).

### 2.3. mRNA Expression Analysis by RT q-PCR

As RNAseq is less accurate for quantifying genes with low expression levels and GO term enrichment analysis does not show detailed changes in expression levels of each constitutive gene, we further evaluated mRNA gene expression levels by qPCR, which has a high quantitative accuracy, for the aforementioned total RNA extracts. cDNA from the total RNA solutions was synthesized with a reverse transcriptase (PrimeScript RT Reagent Kit, TAKARA, Shiga, Japan). A real-time PCR analysis was carried out using a Rotor-Gene 6200 apparatus (Qiagen, Tokyo, Japan). The methods employed for PCR analysis were the same as those by Toyoda et al. [14]. Primers and TaqMan probes used in the present study are listed in Supplementary Table S1. Optimal primers and probes were designed with freely available online tools (https://primers.neoformit.com/; accessed on 12 May 2022). The relative expression levels of the mRNAs were calculated by the comparative Ct method. The amount of target relative to housekeeping mRNA (β-actin) was determined for comparison. All genes analyzed for mRNA levels are listed in Supplementary Table S2.

### 2.4. 16S rRNA Gene Sequencing for Microbiota Analysis

Bacterial DNA in small intestinal and cecal digestas was extracted as previously described [10]. DNA libraries were prepared using a 2-step tailed PCR method. The V3-4 regions of the 16S rRNA gene in each sample were amplified using the forward 341F (ACACTCTTTCCCTACACGACGCTCTTCCGATCT-NNNNN-CCTACGGGNGGCWGCA) and the reverse 805R (TGACTGGAGTTCAGACGTGTGCTCTTCCGATCT-NNNNN-GACTACHVGGGTATCTAATCC) primers for the first PCR analysis, and the forward 2ndF (AATGATACGGCGACCACCGAGATCTACAC-Index2-ACACTCTTTCCCTACACGACGC) and the reverse 2ndR (CAAGCAGAAGACGGCATACGAGAT-Index1-GTGACTGGAGTTCAGACGTGTG) primers for the second PCR analysis. Sequencing was conducted using a MiSeq sequencer with MiSeq Reagent kits v2 (Illumina, SanDiego, CA, USA). The sequences generated from the MiSeq platform were analyzed using the open-source software package Quantitative Insights Into Microbial Ecology 2 (April 2021) [15]. In addition, a SILVA 138 database (https://www.arb-silva.de/; accessed on 7 June 2021) was used to annotate the taxonomic information. Only bacterial taxa, whose mean abundances in the small intestinal and cecal digestas of broilers exceeded 0.1% were used for statistical and correlational analyses. In addition, the bacterial taxa showing significant differences or differential trends, and the bacterial genera found to be associated with the host’s markers, are shown at the result Section 3.3. To take into account differences in the sampling methodology, α-diversity, representing the observed phylogenetic diversity (PD), was estimated using 10,000 randomly selected sequences.

### 2.5. Statistical Analysis

As bacterial abundances are relative values, the Kruskal–Wallis (KW) test, a nonparametric analysis method, was used to analyze the parameters of the relative abundances of each bacterial group. Steel’s test was used for multiple comparisons. Again, as the relative abundance of bacterial taxa was one of the correlation factors in the correlation analysis, the correlation coefficients between the parameters of the abundances of bacterial genera and the pathogenic scores were further analyzed by Spearman’s rank correlation coefficient, a nonparametric analysis. The same methods were used for the other parameters.

Differences between values were considered significant if *p* < 0.05, and with a tendency to be significant if *p* < 0.10. For all the statistical analyses, the values are expressed as the means ± the standard deviations. All statistical analyses were conducted using R software version 4.1.2 (R Core Team, Vienna, Austria).

## 3. Results

### 3.1. RNA Sequencing Analysis

**Figure 1:**
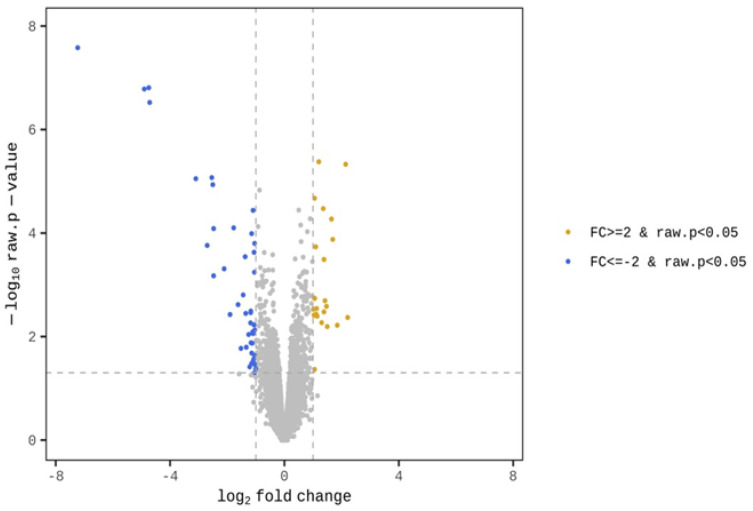
Transcriptional profiles in the duodenal samples of WC-supplemented broilers and control broilers.

**Figure 2:**
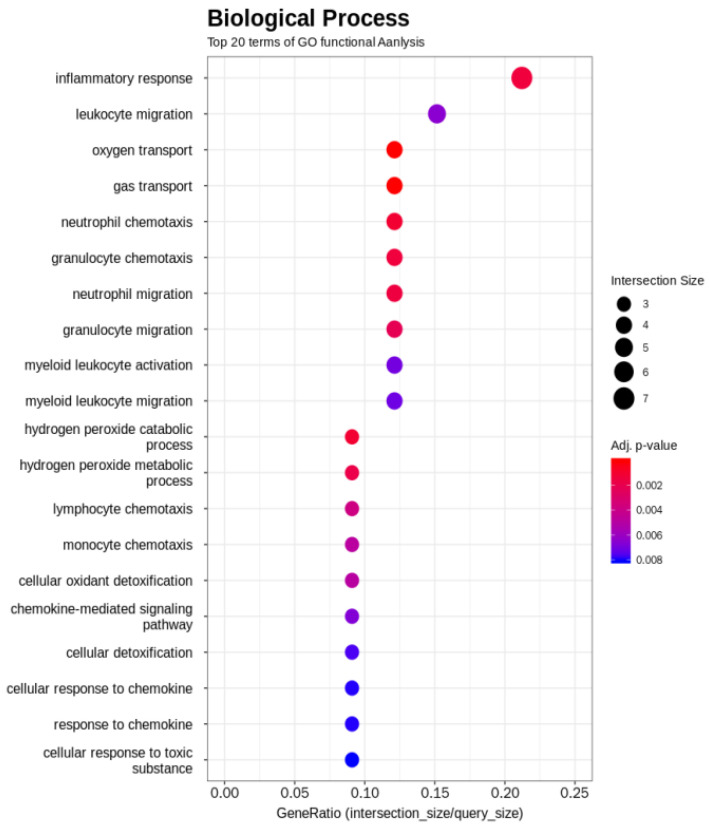
Significant biological processes detected by Gene Ontology term enrichment analysis of WC-supplemented and control broiler samples.

The GO term enrichment analysis was used to interpret the functions of the gene detected in the duodenal samples of broilers. Unlike broilers in the control group, those supplemented with WC had higher and lower expression levels of 23 and 43 genes, respectively (Figure 1). The GO functional analysis also showed an enrichment of the inflammatory response genes (Figure 2). On the one hand, GO functional groups such as oxygen transport, gas transport, hydrogen peroxide catabolic process, hydrogen peroxide metabolic process, cellular oxidant detoxification, cellular detoxification and cellular response to toxic substance were lower in WC-supplemented broilers, compared with the control group. On the other hand, other functional groups such as monocyte chemotaxis, neutrophil chemotaxis, inflammatory response and chemokine-mediated signaling pathway were higher in those broilers treated with WC, again, when compared with control broilers. Indeed, the GO analysis found changes in several genes between the duodenal samples of broiler groups (Table S2), of which, those associated with the expression of inflammatory and defense responses, as well as anti-inflammatory activities, were statistically significant (Figure 2). Upon detecting changes in the gene expression levels of CCL17, CCL18, IL8L2 and SOCS3 cytokines associated with the activity of Th17 and M1/M2 macrophages, it was theorized that WC supplementation may have also been associated with the activity of these immune cells. Therefore, to investigate the changes of these cytokines in detail, we conducted a gene expression analysis by RT-qPCR of Th17 and M1/M2 macrophages associated genes.

### 3.2. Gene Expression Analysis

Duodenal tissue samples were used for the gene expression analysis of cytokines associated with the activity of Th17 and M1/M2 macrophages. The analysis results of the effects of WC supplementation and AM administration on the gene expression in the duodenal samples of broilers are shown in Table 1. The gene expressions of *IL17A*, *IL17F* and *IL22*, which are expressed by Th17 cells, were upregulated by the WC supplementation. The gene expressions of *IL10* and *TGFβ3*, expressed by M2 macrophages, *CATH2*, a factor involved in the production of the antibacterial peptide cathelicidin, *pIgR*, a factor involved in the production of immunoglobulin A (IgA), and *IFNγ*, were also significantly higher in the WC-supplemented group than in the control group. Similarly, the gene expression of *CLDN1*, which is associated with tight junctions, was more than two-fold higher in the WC-supplemented group than in the control group, although it was non-statistically different. Nonetheless, upon conducting a *t*-test analysis between the WC-supplemented and the control groups, significantly statistical differences were observed (*p* = 0.02). The gene expressions of *OCLN*, *ZO-1* and *IL1β* did not differ significantly between the groups.

**Figure 3:**
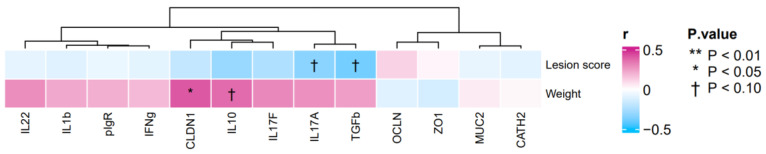
Heatmap of the correlation between gene expression profiles of duodenum, and body weights and small intestinal lesion scores in broiler samples.

The heatmap generated from the gene expression analysis showed positive correlations between body weights and the gene expression of *CLDN1* (r = 0.41, *p* < 0.05) and *IL10* (r = 0.37, *p* = 0.06) (Figure 3). By contrast, the heatmap showed negative correlations between lesion scores and the gene expressions of *IL17A* (r = −0.33, *p* = 0.09) and *TGFβ3* (r = −0.38, *p* = 0.05) (Figure 3).

### 3.3. Microbiota Analysis

The results of the microbiota analysis of the small intestinal and cecal digestas at phylum level are listed in Tables S3 and S4. Beta-diversity is also shown in Figure S1. The microbiota compositions at genus level in the small intestinal digesta are shown in Table 2. The abundances of *Aliicoccus*, *Enterococcus* and *Streptococcus* were significantly greater in the AM-administered group than in the control group (*p* < 0.05). In particular, the abundance of *Alistipes* was 0.23% non-significantly greater in the small intestinal digestas of the WC-supplemented group, than in the control group. Likewise, the abundances of genera *Blautia*, *Esherichia-Shigella* and *Turicibacter* tended to be greater in the AM-administered group than in the control group (*p* < 0.10). No statistical significances were observed in the abundances of genera *Bacteriodes*, *Butyricicoccus*, *Negativibacillus* and *Oscillospira*. The abundance of genus *Aerosphaera* tended to be greater in the small intestinal digestas of AM-administered broilers than in those of the other birds. By contrast, the abundance of *Chloroplast* tended to be lower in the small intestinal digestas of WC-supplemented broilers than in those of the other birds.

The results of the bacterial microbiota analysis of cecal digestas at genus level are shown in Table 3. *Streptococcus* and *Butyricicoccus* had lower abundances (*p* < 0.05), and genus *Oscillospira* tended to have a lower abundance in the samples of AM-administered broilers than in those of control and WC-supplemented broilers. In contrast, *Lachnoclostridia* and genera *Blautia* and *Oscillibacter* had greater abundances (*p* < 0.05), and *Bacteriodes*, *Turicibacters*, *Deitzia* and *Caproiciproducens* tended to be more abundant in the cecal digestas of AM-administered birds, than in those of the other broilers. The genus *Alistipes* tended to be more abundant in the cecal digestas of WC-supplemented broilers, when compared with those of the other birds. Conversely, genus *Lachnospira* tended to be less abundant and *Anaerotruncus* had a significantly lower abundance (*p* < 0.05) in the cecal digesta samples of WC-supplemented broilers than in those of the other birds. Genera *Colidextribacters* and *Flavonifractors* had greater abundances (*p* < 0.05) in the cecal digesta samples of both WC-supplemented and AM-administered groups than in those of the control group. Interestingly, while *Enterococcus* was more abundant (*p* < 0.05) in the samples of WC-supplemented broilers, it had its lowest abundance (*p* < 0.05) in those of AM-administered broilers. Genera *Bacillus* and *Tuzzerella* tended to have lower abundances, and were more abundant (*p* < 0.05) in the cecal digesta samples of WC-supplemented and AM-administered birds, than in those of control broilers. In addition, when compared with that in the control group, the abundance of *Escherichia-Shigella* was greater (*p* < 0.05) in the small intestinal samples of AM-administered broilers.

**Figure 4:**
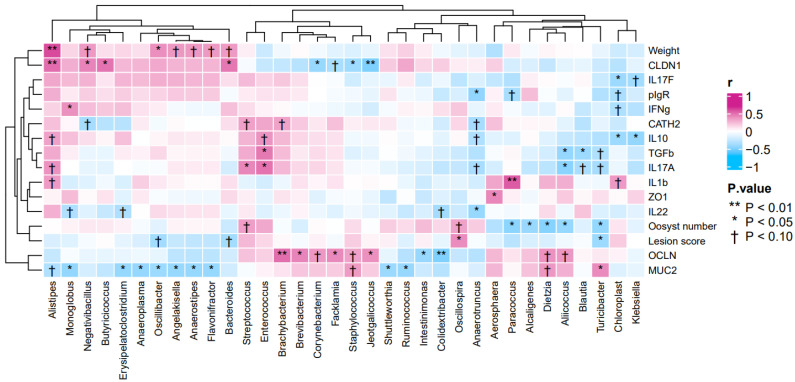
Heatmap of the correlation between gene expression profiles, body weights, lesion scores of broilers and the abundance of bacterial genera in broiler samples.

The association between the gene expression profiles of duodenal tissues, body weights, lesion scores and the abundances of bacterial genera in the small intestines were analyzed by Spearman’s rank correlations (Figure 4). The heatmap showed positive correlations between body weights and the abundance of genus *Alistipes* (r = 0.61, *p* < 0.05), as well as between the gene expression level of *CLDN1* and the abundance of genera *Alistipes* (r = 0.51, *p* < 0.05), *Negativibacillus* (r = 0.44, *p* < 0.05), *Bacteroides* (r = 0.48, *p* < 0.05) and *Butyricicoccus* (r = 0.49, *p* < 0.05). In addition, the heatmap also showed positive correlations between the gene expression level of *IL17A* and the abundances of genera *Streptococcus* (r = 0.43, *p* < 0.05) and *Enterococcus* (r = 0.49, *p* < 0.05). By contrast, the heatmap showed negative correlations between lesion scores and the abundance of genera *Turicibacter* (r = −0.40, *p* < 0.05) and *Oscillospira* (r = 0.41, *p* < 0.05). Other negative correlations found were between the gene expression of *IL17A* and the abundance of genus *Aliicoccus* (r = −0.46, *p* < 0.05), as well as between the gene expression of *TGFβ3* and the abundances of genera *Aliicoccus* (r = −0.43, *p* < 0.05), *Blautia* (r = −0.40, *p* < 0.05) and *Enterococcus* (r = 0.46, *p* < 0.05).

**Figure 5:**
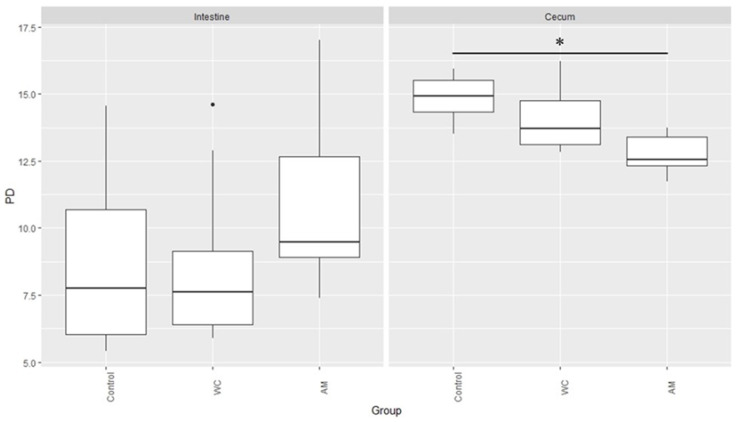
Microbial phylogenetic diversity in the small intestinal and cecal digesta samples of broilers. PD: Phylogenetic Diversity. The asterisk indicates significant differences between control and AM (*p* < 0.05), and dot means outlier.

Lastly, the PD index of the microbiotas in the cecal digesta samples of AM-administered broilers was the lowest, when compared with those of the other broilers (Figure 5).

## 4. Discussion

After detecting changes in the gene expression levels of *CCL17*, *CCL18*, *IL8L2* and *SOCS3*, associated with the activity of Th17 and M1/M2 macrophages, we conducted a further expression analysis by RT-qPCR of genes associated with these macrophages. When compared with those of control broilers, our gene expression analysis detected increases in the expressions of *IL17A*, *IL17F* and *IL22*, which are cytokines produced by Th17 cells, in the small intestinal samples of WC-supplemented broilers (Table 1). Guo et al. [16] observed that healthy broilers supplemented with WC had a higher expression of *IL17* in their jejunum than control broilers did. Similarly, WC strain sc-09 was shown to upregulate the gene expression of *IL22* in B cells when incubated with murine splenocytes, in a greater manner than other lactic acid bacteria did [17]. These reports indicate that supplementation of WC activates Th17 cells, which is consistent with the results of the present study. Moreover, a negative correlation was observed between lesion scores and gene expression level of *IL17A* (Figure 3), suggesting that *IL17* may contribute to the improvement of tissue damage. The association of an upregulated *IL17* gene expression of the small intestine, with the decrease in the number of oocysts and improved lesion scores, has been reported earlier [18]. In addition, it has been shown that a IL17R^−/−^ murine model was more vulnerable to toxoplasmosis than wildtype mice [19]. *IL17A* and *IL22* have been proven to recruit neutrophils and to increase the gene expression of anti-microbial peptides *RegIIIβ* and *RegIIIγ* in ceca of mice challenged with *Eimeria falciformis* [20]. More interestingly, the presence of *IL17A* and *IL22* inhibited the parasitic growth of *E. falciformis*, both in vivo and in vitro [20]. At these premises, we previously found that WC supplementation helped augment the body weights of coccidium-infected broilers, while at the same time helped lower the numbers of intestinal oocysts and improve lesion scores [10]. This evidence seems to imply that an upregulated gene expression of *IL17* in the duodenal mucosa could help inhibit the development and the pathogenic effects of coccidiosis, and even perhaps improve production performance parameters such as weight gain.

In the present study, the gene expression level of *CATH2*, which is a well-established antimicrobial peptide encoding mRNA [21], was higher in WC-supplemented broilers than in control broilers (Table 1). *IL17*/*IL22* stimulation reduces the numbers of *E. falciformis* through the enhancement of antimicrobial peptides production [20]. Our analysis also found a greater expression of *pIgR* in WC-supplemented broilers, when compared with the control group (Table 1). *pIgR* transcellularly transports IgA, the first line of defense against pathogens, to the intestinal lumen and across the epithelial cells [22]. *IL17* is believed to be involved in optimal IgA production and to regulate the expression of *pIgR* [23]. Thus, our results also seemed to imply that, unlike in control broilers, enhanced intestinal barrier functions, induced by the activity of these cytokines, occurred in WC-supplemented broilers.

The mean gene expression level of *CLDN1*, associated with tight junctions, was around 2.4-fold higher in the WC-supplemented broilers than in the control group. Unfortunately, no significant differences were found for *OCLN* and *ZO-1*. Nonetheless, a *t*-test determined that the difference in *CLDN1* gene expression between WC and control groups was significant, and that *CLDN1* was positively associated with weight gains (Table 1 and Figure 2). The presence of pathogens causes abnormal paracellular permeability and cytotoxicity, resulting in the weakening of tight junction components, especially claudins [24,25]. In turn, the pathogenic effects induce low production levels and high mortality in poultry [26]. However, Kinugasa et al. [27] observed that *IL17* is associated with the enhancement of the tight junction protein expression. Therefore, our results seemed to suggest that WC supplementation, via Th17 cell activation, helped to prevent leaky guts that would otherwise be caused by the coccidial infection.

Our results showed that the gene expressions of *TGFβ3* and *IL10* were significantly upregulated only in WC-supplemented broilers (Table 1). Intestinal macrophages residing in the lamina propria have disparate yet interchangeable functions. For example, while M1 macrophages are generally reported as being pro-inflammatory, M2 macrophages are anti-inflammatory [28,29]. Indeed, M1 macrophages are thought to induce the gene expression of proinflammatory *IL1β* [30], and M2 macrophages are involved in wound healing, angiogenesis, tissue remodeling and the production of anti-inflammatory *IL10* [31] and TGFβ [32]. *IL10* has been reported to help maintain and regenerate the epithelial layer of the small intestine [33]. In addition, Zhang et al. [9] found that WC supplementation reduced the level of *IL1β*, while incrementing the level of *IL10* in the sera of broilers, which is in line with the results of the present study. Therefore, based on the resulting evidence, it can be deduced that WC supplementation was effective in stimulating an anti-inflammatory activity, such as that of *IL10* and *TGFβ3*. Furthermore, *IL10* and *TGFβ3* were likely involved in modulating the inflammation caused by the coccidial infection in broilers of the present work.

Upon analysis of the microbiotas and when compared with those of control and AM-administered birds, it was established that the genus *Alistipes* had its highest, although not statistically significant, abundance in the small intestinal digestas of WC-supplemented broilers (Table 2). Similarly, the abundance of *Alistipes* tended to be higher (*p* = 0.073) in the cecal digestas of WC-supplemented birds, when compared with those of control broilers (Table 3). Moreover, the results of gene expression analysis in the small intestine and its association with the microbial composition revealed that positive correlations were observed between the abundance of *Alistipes*, body weights and the gene expression level of *CLDN1* (Figure 3). In a separate study, a high abundance of *Alistipes* was observed in the microbiotas of broilers with greater body weights than in those with lower body weights [34,35,36,37,38]. Supplementation with WC was also reported to be associated with a high abundance of *Alistipes*, which in turn, reportedly induced increased weight gain and *IL10* expression, as well as enhanced tight junction functions [34,35,36,37,38]. Our results are in full agreement with these anteceding reports. Yet, a more encouraging outcome was the fact that the abundance of *Enterococcus* either tended to be greater or was significantly greater in the samples of WC-supplemented birds, but not in those of control broilers. Moreover, the abundance of *Enterococcus* and the gene expression of *IL17A* and body weight were positively correlated in a significant manner (Figure 3 and Figure 4). Thus, it can be reasoned that *Enterococcus* could have induced weight gain via the enhancement of *IL17A*, as was the case with *Alistipes*. Enterococci are largely known as beneficial bacteria for poultry and hence, it was unsurprising that in the present work, the dominance of *Enterococcus*, along with an increase in the gene expression of *IL17A*, was associated with increased weight gain.

Interestingly, when compared with that in the other birds, the abundance of *Esherichia-Shigella*, either tended to be greater in the small intestinal digestas (Table 2) or was significantly greater in the cecal digestas (Table 3) of AM-administered birds. In a previous study, we showed that *E. coli* numbers were higher in the cecal digestas of AM-administered broilers than in those of control birds [10]. The results of the present study all but validated our previous report. Indeed, increases in the number of bacteria belonging to the family Enterobacteriaceae (e.g., *Esherichia-Shigella*) is considered an indicator of dysbiosis [39]. In the present study, the PD index was lower in a significant manner in the cecal microbiotas of AM-administered birds, than in those of the control broiler (Figure 5; *p* < 0.05). Antimicrobials are known to disrupt the composition of the intestinal microbiota [40] and to delay the maturation of the intestinal microbiota of broilers [41]. Feed-additive probiotics, on the other hand, have been shown to accelerate the microbiota maturation [41], which is consistent with our results. Finally, it has been reported that dysbiosis induced by antibiotics reduces the intestinal epithelial barrier functions [13,42,43]. This past work also seemed to suggest that the administration of anticoccidial agents could disrupt the intestinal microbiota. An altered microbiota likely reduces the intestinal barrier functions and hence, prevents AM-administered broilers from gaining weight [10].

## 5. Conclusions

In the present work, it was demonstrated that the administration of AM to broilers caused a lower PD in the intestinal microbiotas, resulting in the overabundance of opportunistic and potential pathogens, and the underabundance of bacteria known to be beneficial. In contrast, WC supplementation increased the abundance of beneficial bacteria, such as genera *Alistipes* and *Enterococcus*, and upregulated the gene expression levels of *IL17A*, *IL17F* and *IL22*, cytokines produced by Th17 cells, and *IL10* and *TGFβ3*, via the anti-inflammatory M2 macrophages. All of this probably resulted in more robust intestinal barrier functions and increased gene expression levels of *CATH2*, *CLDN1* and *pIgR*. As a result, these beneficial effects exerted by WC supplementation on broilers likely helped to lower lesion scores and increase weight gain, while at the same time ameliorating the coccidial symptoms and lowering the *Eimeria* oocyst numbers. What is more, genera *Alistipes* and *Enterococcus* plausibly contributed to the overall immunostimulatory effect. To conclude, the results of the present work showed that WC strain SANK70258 supplementation induced weight gain through a specific immunostimulatory mechanism, without disrupting the intestinal microbiota composition, as coccidiostat lasalocid-A sodium administration did.

## Figures and Tables

**Table 1:** Gene expression levels of WC and AM in Th17 cells, macrophages and the barrier function in the small intestinal samples of broilers.

Gene	Control	WC	AM	KW *p* Value ‡
Anti-microbial peptide-associated gene				
*CATH2*	1.76 ± 2.64	16.39 ± 10.90 *	1.57 ± 1.43	<0.05
Tight junction-associated genes				
*CLDN1*	1.33 ±0.91	3.16 ± 1.99	2.35 ± 1.07	0.110
*OCLN*	1.05 ± 0.33	1.29 ± 0.36	1.16 ± 0.32	0.416
*ZO-1*	1.01 ± 0.17	1.30 ± 0.38	1.42 ± 0.75	0.289
IgA-associated genes				
*pIgR*	2.55 ± 1.61	8.89 ± 5.31 *	4.79 ± 4.17	<0.05
M1 macrophage-associated genes				
*IFNγ*	1.90 ± 1.31	3.82 ± 1.73 *	4.13 ± 4.53	0.061
*IL1β*	1.30 ± 1.36	1.36 ± 0.80	1.34 ± 0.55	0.403
M2 macrophage-associated genes				
*IL10*	1.77 ± 1.25	19.91 ± 12.01 *	2.49 ± 1.91	<0.05
*TGFβ3*	1.03 ± 0.26	1.78 ± 0.55 *	1.03 ± 0.31	<0.05
Th17 cell-associated genes				
*IL17A*	1.55 ± 1.39	4.63 ± 2.07 *	1.17 ± 0.81	<0.05
*IL17F*	10.18 ± 24.23	30.46 ± 28.91 *	13.28 ± 16.02	<0.05
*IL22*	1.23 ± 0.80	2.24 ± 0.79 *	2.71 ± 2.33	0.081

All parameters are shown as the means ± the standard deviations (*n* = 9). Control: non-treatment control. WC: supplementation with 3 × 10^5^ CFU/g of *W. coagulans* strain SANK70258. AM: 75 ppm of sodium lasalocid-A in the feed. The asterisks indicate significant differences between control and WC or AM (*p* < 0.05). The double dagger indicates the probability value in the Kruskal–Wallis test.

**Table 2:** Relative abundances of bacteria at genus level in the small intestinal digestas of broilers.

Taxon	Control	WC	AM	KW *p* Value ‡
*Aerosphaera*	0.00 ± 0.00	0.03 ± 0.09	0.26 ± 0.40 †	<0.05
*Aliicoccus*	0.11 ± 0.19	0.09 ± 0.19	0.66 ± 0.56 *	<0.05
*Alistipes*	0.00 ± 0.00	0.23 ± 0.63	0.00 ± 0.01	0.134
*Bacteroides*	1.62 ± 2.33	3.41 ± 6.39	4.19 ± 7.47	0.346
*Blautia*	0.05 ± 0.14	0.01 ± 0.03	0.24 ± 0.30 †	<0.05
*Butyricicoccus*	0.78 ± 1.17	0.67 ± 0.96	0.61 ± 1.04	0.951
*Chloroplast*	0.36 ± 0.18	0.21 ± 0.42 †	0.26 ± 0.34	0.066
*Clostridium* *_sensu_stricto_1*	0.80 ± 0.94	0.33 ± 0.28	0.12 ± 0.21*	<0.05
*Dietzia*	0.81 ± 0.73	0.78 ± 0.46	1.46 ± 0.57 †	<0.05
*Enterococcus*	8.83 ± 3.70	12.12 ± 6.68	4.74 ± 4.21 *	<0.05
*Escherichia-Shigella*	3.89 ± 5.59	4.97 ± 4.67	11.93 ± 15.10 †	0.114
*Negativibacillus*	0.25 ± 0.46	0.27 ± 0.43	0.34 ± 0.63	0.969
*Oscillospira*	0.06 ± 0.16	0.00 ± 0.00	0.00 ± 0.00	0.325
*Streptococcus*	8.80 ± 4.04	14.81 ± 8.31	3.87 ± 6.71 *	<0.05
*Turicibacter*	3.37 ± 3.53	1.59 ± 1.70	8.38 ± 6.05 †	<0.05

All parameters are shown as the means ± the standard deviations (*n* = 9). Control: non-treatment control. WC: supplementation with 3 × 10^5^ CFU/g of *W. coagulans* strain SANK70258. AM: 75 ppm of sodium lasalocid-A in the feed. The asterisks indicate significant differences between control and WC or AM (*p* < 0.05). A dagger indicates a differential trend between control and WC or AM (*p* < 0.1). The double dagger indicates the probability value in the Kruskal–Wallis test.

**Table 3:** Relative abundances of bacteria at genus level in the cecal digestas of broilers.

Taxon	Control	WC	AM	KW *p* Value ‡
*Alistipes*	0.00 ± 0.00	1.03 ± 1.73 †	0.01 ± 0.02	0.073
*Anaerotruncus*	0.53 ± 0.63	0.09 ± 0.10 *	0.60 ± 0.58	<0.05
*Bacillus*	7.76 ± 4.34	3.39 ± 2.66 †	2.28 ± 2.26 *	<0.05
*Bacteroides*	6.29 ± 7.67	12.48 ± 10.72	16.45 ± 10.36 †	0.088
*Blautia*	0.22 ± 0.34	0.18 ± 0.20	1.04 ± 0.68 *	<0.05
*Butyricicoccus*	5.13 ±2.17	4.92 ± 1.80	2.07 ±1.09 *	<0.05
*Caproiciproducens*	0.10 ± 0.10	0.13 ± 0.14	0.18 ± 0.07 †	0.168
*Colidextribacter*	1.31 ± 0.46	0.42 ± 0.50 *	0.26 ± 0.34 *	<0.05
*Dietzia*	0.00 ± 0.01	0.00 ± 0.01	0.03 ± 0.04 †	<0.05
*Enterococcus*	1.27 ± 0.37	1.95 ± 0.61 *	0.13 ± 0.13 *	<0.05
*Escherichia-Shigella*	0.75 ± 0.55	1.47 ± 1.69	4.87 ± 3.36*	<0.05
*Flavonifractor*	0.02 ± 0.05	0.15 ± 0.13 *	0.28 ± 0.41 *	<0.05
*Lachnoclostridium*	0.35 ± 0.22	0.59 ± 0.47	1.21 ± 0.66 *	<0.05
*Lachnospira*	0.11 ± 0.03	0.05 ± 0.10 †	0.08 ± 0.05	0.057
*Oscillibacter*	0.12 ± 0.16	0.24 ± 0.23	0.54 ± 0.52 *	<0.05
*Oscillospira*	0.27 ± 0.32	0.07 ± 0.19	0.01 ± 0.03 †	0.072
*Streptococcus*	1.29 ± 0.26	1.24 ± 0.85	0.28 ± 0.40 *	<0.05
*Turicibacter*	0.13 ± 0.08	0.23 ± 0.26	0.76 ± 0.29 †	<0.05
*Tuzzerella*	3.42 ± 2.21	1.55 ± 1.80 †	0.25 ± 0.39 *	<0.05

All parameters are shown as the means ± the standard deviations (*n* = 9). Control: non-treatment control. WC: supplementation with 3 × 10^5^ CFU/g of *W. coagulans* strain SANK70258. AM: 75 ppm of sodium lasalocid-A in the feed. The asterisks indicate significant differences between control and WC or AM (*p* < 0.05). A dagger indicates a differential trend between control and WC or AM (*p* < 0.1). The double dagger indicates the probability value in the Kruskal–Wallis test.

## Data Availability

Data are available from the corresponding author.

## Supplementary Materials

The following supporting information can be downloaded at: https://www.mdpi.com/article/10.3390/pathogens12010096/s1, Table S1: List of analyzed genes and used primers and probes; Table S2: The result of term_id and DEG analysis summarized based on genes; Table S3: Relative abundances of bacteria at phylum level in the small intestinal digestas of broilers; Table S4: Relative abundances of bacteria at phylum level in the cecal digestas of broilers; Figure S1: Beta diversity (weighted unifrac PCoA) comparisons between the data from control (C), *W. coagulans* strain SANK70258 (WC)-supplemented and lasalocid-A sodium (AM)-administered broilers.
