# Supplementary material for: Dietary Weizmannia coagulans Strain SANK70258 Ameliorates Coccidial Symptoms and Improves Intestinal Barrier Functions of Broilers by Modulating the Intestinal Immunity and the Gut Microbiota
Source: Pathogens. 2023 Jan 6;12(1):96. doi: 10.3390/pathogens12010096 (PMC9864622; doi:10.3390/pathogens12010096)
Supplement: Supplementary file 1 [file pathogens-12-00096-s001.zip › Supplemental files/FigureS1_fin.pptx]

## Slide 1
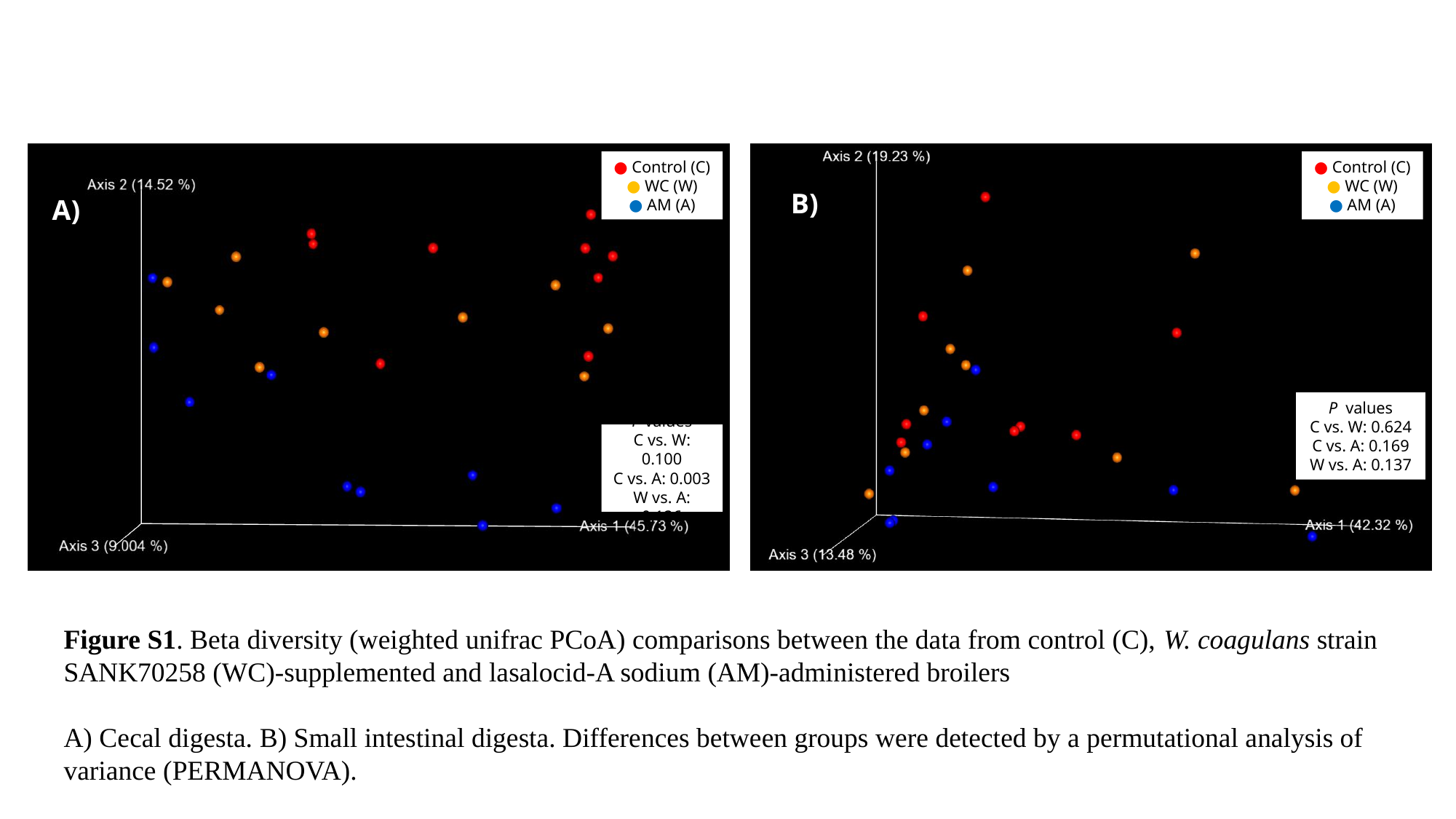

● Control (C)
● WC (W)
● AM (A)
● Control (C)
● WC (W)
● AM (A)
B)
A)
P values
C vs. W: 0.624
C vs. A: 0.169
W vs. A: 0.137
P values
C vs. W: 0.100
C vs. A: 0.003
W vs. A: 0.126
Figure S1. Beta diversity (weighted unifrac PCoA) comparisons between the data from control (C), W. coagulans strain SANK70258 (WC)-supplemented and lasalocid-A sodium (AM)-administered broilers
A) Cecal digesta. B) Small intestinal digesta. Differences between groups were detected by a permutational analysis of variance (PERMANOVA).
